# Transpiration response to soil drying versus increasing vapor pressure deficit in crops: physical and physiological mechanisms and key plant traits

**DOI:** 10.1093/jxb/erad221

**Published:** 2023-06-24

**Authors:** Tina Koehler, Fabian J P Wankmüller, Walid Sadok, Andrea Carminati

**Affiliations:** Physics of Soils and Terrestrial Ecosystems, Department of Environmental Systems Science, ETH Zurich, Zurich, Switzerland; Soil Physics, Bayreuth Center of Ecology and Environmental Research (BayCEER), University of Bayreuth, Bayreuth, Germany; Physics of Soils and Terrestrial Ecosystems, Department of Environmental Systems Science, ETH Zurich, Zurich, Switzerland; Agronomy and Plant Genetics, College of Food, Agricultural and Natural Resource Sciences, University of Minnesota, Twin Cities, MN, USA; Physics of Soils and Terrestrial Ecosystems, Department of Environmental Systems Science, ETH Zurich, Zurich, Switzerland; University of Florida, USA

**Keywords:** Plant traits, root water uptake, soil drying, soil–plant hydraulics, stomatal regulation, transpiration rate, vapor pressure deficit

## Abstract

The water deficit experienced by crops is a function of atmospheric water demand (vapor pressure deficit) and soil water supply over the whole crop cycle. We summarize typical transpiration response patterns to soil and atmospheric drying and the sensitivity to plant hydraulic traits. We explain the transpiration response patterns using a soil–plant hydraulic framework. In both cases of drying, stomatal closure is triggered by limitations in soil–plant hydraulic conductance. However, traits impacting the transpiration response differ between the two drying processes and act at different time scales. A low plant hydraulic conductance triggers an earlier restriction in transpiration during increasing vapor pressure deficit. During soil drying, the impact of the plant hydraulic conductance is less obvious. It is rather a decrease in the belowground hydraulic conductance (related to soil hydraulic properties and root length density) that is involved in transpiration down-regulation. The transpiration response to increasing vapor pressure deficit has a daily time scale. In the case of soil drying, it acts on a seasonal scale. Varieties that are conservative in water use on a daily scale may not be conservative over longer time scales (e.g. during soil drying). This potential independence of strategies needs to be considered in environment-specific breeding for yield-based drought tolerance.

## Teaser

A review explaining the link between soil–plant hydraulics and water use regulation during soil and atmospheric drying.

## Introduction

Drought events are predicted to become more frequent globally as part of the overall global atmospheric drying (Dai *et al.*, 2018; Yuan *et al.*, 2019) due to increased temperatures and changes in the hydrological cycle (IPCC, 2022). The resulting rise in atmospheric evaporative demand—vapor pressure deficit (VPD, kPa)—is one of the main drivers of plant water deficit as it impacts evapotranspiration from soils and plants (Jung *et al.*, 2010; Novick *et al.*, 2016; Dai *et al.*, 2018). The vapor pressure deficit is the difference in water vapor pressure in the leaves, which is assumed to be at saturation (*e*_s_, kPa) at air temperature, and the atmospheric water vapor pressure (*e*_a_, kPa) for a given temperature:


$$\begin{array}{l} \text{VPD}=e_{\text{s}}-e_{\text{a}}. \end{array}$$ (1)

In combination with more variable rainfall patterns and depending on soil hydraulic properties, the enhanced atmospheric water demand may further lead to more frequent and severe limitations in soil water availability, which is the second main driver of plant water deficit.

From a plant’s perspective, water deficit occurs when water availability cannot match plant water demand for growth and transpiration at a particular time (Begg and Turner, 1976; Draye *et al.*, 2010; Tardieu *et al.*, 2018). When extending over long periods, demand for water that exceeds the supply typically leads to a decline in transpiration and growth rates, which translates into lower grain yields (Reynolds *et al.*, 1994; Lobell and Gourdji, 2012; López *et al.*, 2021). Yield is particularly affected when water deficit builds up over time and negatively affects key yield-making phenological phases in the cropping cycle, particularly the reproductive stage (Sinclair and Muchow, 2001; Sinclair *et al.*, 2005, 2010). In light of a growing food demand worldwide, it is therefore essential to understand and potentially predict plant transpiration response to the drivers of drought that affect the water supply (soil moisture) and water demand (VPD).

As a result of rising VPD, the increasing gradient of vapor pressure between the relatively dry atmosphere and the moist inner space of a leaf is expected to drive larger transpiration rates. Transpiration from the leaves creates a suction on the water column within the leaf xylem, which drives the water flow from the soil to the roots along a gradient of water potentials within the soil–plant–atmosphere continuum (SPAC). The flow is proportional to the differences in water potentials and the hydraulic conductance of the components of the SPAC (Cowan, 1965, 1972). Root water uptake is determined by transpiration at the leaf level and must compensate for the water loss. If supply does not meet demand, the plant will eventually wilt. Plant transpiration rate (Tr, mg s^−1^) is driven by VPD relative to atmospheric pressure (*P*_atm_, kPa) and depends on leaf area (LA, cm²). Additionally, on a short time scale, transpiration regulation is related to the stomatal conductance to water vapor (*g*_sw_, mg s^−1^ cm^−2^) of the leaves and can be expressed as (Buckley, 2019; Brodribb *et al.*, 2020):


$$\begin{array}{l} \text{Tr}\;=\;\frac{\text{VPD}}{P_{\text{atm}}}\;\times \;\text{LA}\;\times \;g_{\text{sw}}. \end{array}$$ (2)

Two main groups of mechanisms have been demonstrated to be involved in stomatal regulation: firstly, metabolic mechanisms that operate through the production of hormones, including abscisic acid (ABA; Assmann and Jegla, 2016; Sussmilch and McAdam, 2017; Sussmilch *et al.*, 2017) and auxins (indole-3-acetic acid, IAA; Péret *et al.*, 2012; Sadok and Schoppach, 2019). Such phytohormones are believed to mediate stomatal closure by regulating the plant hydraulic conductance (see next paragraph), e.g. by the activation of aquaporins (AQP, water-channeling proteins that facilitate the transport of water molecules across biological membranes; e.g. Johansson *et al.*, 2000; Li *et al.*, 2014) in different parts of the plant (e.g. Sadok and Sinclair, 2010; Pantin *et al.*, 2013; Schoppach *et al.*, 2014). Moreover, ABA was shown to induce stomatal closure by having a direct biochemical effect on guard cells, causing a change in the osmotic potential of guard cells (Buckley, 2019).

Secondly, a passive mechanism of stomatal closure induced by the hydraulic connection between epidermal cells and guard cells was demonstrated (Buckley *et al.*, 2003). Sperry and Love (2015) and Carminati and Javaux (2020) have suggested a hydraulic framework to explain the observation that stomatal conductance is connected to leaf water status (i.e. leaf water potential; Anderegg *et al.*, 2017) by linking hydraulic constraints on transpiration and stomatal regulation. They postulated that transpiration becomes constrained by soil–plant hydraulics when the resistance in some parts of the SPAC increases to the extent that leaf water potential starts to decline non-linearly with increasing transpiration rate. The premise is that stomatal regulation avoids the disproportionate drop in leaf water potential by responding to non-linearities in the slope of the relationship between leaf water potential and transpiration rate (i.e. to a decrease in soil–plant hydraulic conductance):


$$\begin{array}{l} K_{\text{sp}}=\frac{\text{Tr}}{\left({\;\;\;\psi \;\;\;}_{\text{soil }\;\;\;\;\;\;}-\;{\;\;\;\psi \;\;\;}_{\text{leaf}}\right)}, \end{array}$$ (3)

where *K*_sp_ (mg s^−1^ kPa^−1^) is the soil–plant hydraulic conductance, Tr (mg s^−1^) is the transpiration rate, and ψ_soil_ (kPa) and ψ_leaf_ (kPa) are the soil matric and leaf water potential, respectively. The physiological mechanism by which plants can sense a change in hydraulic conductance, and not simply a change in water status (e.g. leaf water potential or leaf turgidity), is not clear. Wankmüller and Carminati (2022) hypothesize that a change in hydraulic conductance could be sensed by plants via ABA biosynthesis (related to a decline in leaf water potential and turgidity; e.g. McAdam and Brodribb, 2018; Merilo *et al.*, 2018) and ABA degradation (related to an increase in carbon assimilation; e.g. Tallman, 2004; Nováková *et al.*, 2005). This would indeed allow plants to down-regulate stomata when the relation between leaf water potential and transpiration becomes non-linear. However, this idea remains largely speculative, particularly regarding the relation between ABA degradation and assimilation. Although the specific physiological mechanisms responsible for stomatal closure are not fully understood, there is substantial empirical evidence indicating the coordination between limitations in *K*_sp_ and stomatal conductance (Rodriguez-Dominguez and Brodribb, 2020; Abdalla *et al.*, 2022). Depending on the environmental conditions (water supply versus demand), the decrease in hydraulic conductance of the SPAC can either be dominated by an increasing resistance to water flow through the plant (e.g. in wet soil and high VPD; Sinclair *et al.*, 2008b; Couvreur *et al.*, 2012) or through the soil (e.g. in dry soil and low VPD; Sinclair *et al.*, 2008a; Carminati and Javaux, 2020). Thus, the traits that can potentially modify the transpiration rate response to atmospheric drying differ from the ones involved in the transpiration rate regulation during soil drying.

One approach for plants to avoid a disproportionate drop in leaf water potential with increasing transpiration is to have a low leaf-level gas exchange during periods of high VPD and soil drying, which may lead to an improved crop water use efficiency and the conservation of soil water under terminal drought by partly closing stomata (Sinclair *et al.*, 2005). However, the question of whether limiting Tr in response to elevated VPD or decreasing soil moisture supply is advantageous for yield highly depends on the dynamics of water demand and supply over the crop cycle (e.g. Sinclair *et al.*, 2010, 2014; Messina *et al.*, 2015; Sadok and Schoppach, 2019). For instance, Messina *et al.* (2015) showed in their simulation study of maize (*Zea mays* L.) that limiting Tr at high VPD may, on the one hand, lead to a yield increase in drought-prone environments, potentially due to soil water conservation early in the growing period (Vadez *et al.*, 2014), or on the other hand, lead to yield penalties in well-watered environments, potentially due to unnecessary limitations on carbon assimilation (Sadok *et al.*, 2019). This context-dependency illustrates the need to separately dissect the physiological and physical mechanisms behind the transpiration rate response to increasing VPD versus decreasing soil moisture to identify the best drought adaptation strategies as a function of the target environmental scenario and, therefore, to inform location-specific breeding strategies better (Hammer *et al.*, 2006; Tardieu *et al.*, 2018) to manage drought risk.

This review analyses the extensive literature on the diversity of crop transpiration rate response to increasing evaporative demand and decreasing soil moisture to point out similarities and differences between plant transpiration response to different drivers of water limitations. To this end, we investigate plant transpiration adaptation strategies (e.g. conservative versus consumptive water use) as a function of the water supply and demand under varying environmental conditions. We use a quantitative hydraulic framework that can theoretically simulate the transpiration rate response to both environmental drivers of drought by linking the observation that stomatal closure is associated with a decrease in leaf water potential to soil–plant hydraulics, in order to explain the observed data. The goal of this review is to identify plant hydraulic traits that impact water use regulation, in the context of improving yield in water limited environments for varying atmospheric and edaphic conditions, rather than to discuss physiological mechanisms of stomatal closure.

## Ranges and diversity of transpiration rate response curves to increasing vapor pressure deficit and decreasing soil moisture in crops

**Fig. 1.**
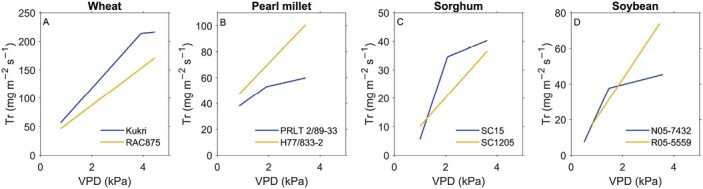
Characteristic response of transpiration rate (Tr) to increasing vapor pressure deficit (VPD) comparing a genotype that increases transpiration rate linearly with increasing VPD (orange) versus a genotype that restricts the increase in transpiration rate at a certain threshold VPD (blue). Redrawn for wheat [Schoppach and Sadok, 2012, with permission from Elsevier, © Elsevier (2012), please note, the Open Access licence covering this article does not apply to this image]; pearl millet (Kholová *et al.*, 2010b); sorghum [Choudhary *et al.*, 2013b; with permission conveyed through Copyright Clearance Center, Inc., © CSIRO Publishing (2013), please note, the Open Access licence covering this article does not apply to this image]; and soybean [Devi *et al.*, 2014; © John Wiley and Sons (2104), please note, the Open Access licence covering this article does not apply to this image], with permission, using WebPlotDigitizer (Rohatgi, 2022).

A large number of studies characterizing the diversity of the transpiration rate response to increasing VPD have been reported in many crop species (Table 1). Typically, the transpiration rate response to increasing VPD is tested in wet soils to disentangle reasons for transpiration rate limitations between atmospheric and soil drying. Usually, rising VPD is experimentally induced by simultaneously varying temperature and relative humidity. It is important to mention that this experimental approach might be flawed as temperature was demonstrated to interact non-linearly with the transpiration rate sensitivity to increasing VPD (Yang *et al.*, 2012; Sadok *et al.*, 2021). Moreover, the transpiration rate response to VPD was shown to vary with VPD conditions during growth (Seversike *et al.*, 2013; Riar *et al.*, 2015; Choudhary *et al.*, 2020). Therefore, comparing results from different studies is hardly possible. However, typical results show a range of possible response curves between two extreme responses (Fig. 1). Some genotypes exhibit a highly consumptive water use pattern, reflected by a linear, high slope increase in the transpiration rate with increasing VPD (yellow curves, Fig. 1). In contrast, other genotypes show a water-saving behavior, with the transpiration rate decreasing its slope past a certain VPD breakpoint (VPD_BP_, kPa, blue curves, Fig. 1). Thereby, VPD_BP_ can vary considerably between genotypes (Table 1). VPD-sensitive genotypes close stomata at relatively low VPD_BP_. Less responsive genotypes transpire linearly until comparatively dry atmospheric conditions (high or no VPD_BP_ within the tested range of VPD).

**Table 1.** Examples of studies reporting crop inter- and intra-specific variations of the transpiration rate response to increasing VPD conditions

	VPD_BP_ (kPa)		Slope 1 (mg H_2_O m^−2^ s^−1^)		Slope 2 (mg H_2_O m^−2^ s^−1^)			
Species	Max.	Min.	Max.	Min.	Max.	Min.	Regression	Reference
Chickpea	2.24	1.69	12.15	8.60	5.51	2.17	Segmented	Anbazhagan *et al.* (2015)
Chickpea	3.07	1.84	79.68	40.01	8.64	−34.17	Segmented	Sivasakthi *et al.* (2017)
Chickpea			36.67	31.67			Linear	Sivasakthi *et al.* (2020)
Chickpea	2.69	2.53	36.67	35.01	−1.67	−18.34	Segmented	
Chickpea			7.18	4.5			Linear	Zaman-Allah *et al.* (2011)
Chickpea	2.55	2.54	11.49	10.77	1.61	−4.69	Segmented	
Cowpea			35.28	24.72			Linear	Belko *et al.* (2012)
Cowpea	2.92	1.81	34.17	23.06	14.72	3.61	Segmented	
Durum wheat			13.90	11.11			Linear	Medina *et al.* (2018)
Durum wheat	1.10	1.05	80.56	30.56	16.67	8.33	Segmented	
Maize			36.20	16.40			Linear	Jafarikouhini *et al.* (2022)
Maize	2.48	1.30	69.50	24.20	—	—	Segmented	
Maize	2.52	1.74	—	—	—	—	Segmented	Choudhary *et al.* (2014)
Maize			10.83	−0.98			Linear	Choudhary *et al.* (2020)
Maize	4.14	3.03	17.69	10.84	1.17	−4.85	Segmented	
Maize			40.70	17.70			Linear	Gholipoor *et al.* (2013a)
Maize	2.52	1.69	—	—	15.10	3.20	Segmented	
Maize	2.19	1.72	20.04	18.01	—	—	Segmented	Yang *et al.* (2012)
Peanut			22.30	—			Linear	Devi and Sinclair (2011)
Peanut	2.25	1.81	35.10	15.80	3.63	−10.10	Segmented	
Peanut			28.50	11.90			Linear	Devi *et al.* (2010)
Peanut	2.56	1.98	33.40	18.40	6.34	−6.27	Segmented	
Peanut	2.90	1.61	55.72	24.52	0.17	−54.44	Segmented	Shekoofa *et al.* (2013)
Peanut			30.80	11.00			Linear	Shekoofa *et al.* (2015)
Peanut	2.80	2.30	16.50	12.20	17.10	−10.70	Segmented	
Pearl millet			16.73	6.86			Linear	Choudhary *et al.* (2020)
Pearl millet	4.35	2.67	11.92	7.91	5.49	−1.31	Segmented	
Pearl millet			59.45	11.39			Linear	Kholová *et al.* (2010b)
Pearl millet	1.91	1.45	50.56	13.61	16.11	4.44	Segmented	
Quinoa			40.77	38.98			Linear	Sanchez *et al.* (2021)
Quinoa	2.40	1.84	67.98	48.90	35.55	−6.82	Segmented	
Sorghum	2.72	1.17	—	—	—	—	Segmented	Choudhary and Sinclair (2014)
Sorghum			11.24	6.62			Linear	Choudhary *et al.* (2013a)
Sorghum	2.91	1.17	40.30	8.96	7.27	−12.49	Segmented	
Sorghum			13.43	10.18			Linear	Choudhary *et al.* (2020)
Sorghum	4.42	3.03	17.72	8.78	7.71	−13.61	Segmented	
Sorghum	2.72	1.62	56.30	30.20	11.30	−6.10	Segmented	Gholipoor *et al.* (2010)
Sorghum			34.90	13.00			Linear	Riar *et al.* (2015)
Sorghum	2.99	2.50	36.60	15.20	13.60	5.56	Segmented	
Sorghum			10.10	8.35			Linear	Shekoofa *et al.* (2014)
Sorghum	3.87	2.33	—	—	—	—	Segmented	
Soybean			21.60				Linear	Devi *et al.* (2014)
Soybean	1.94	1.41	42.60	25.30	8.95	−7.75	Segmented	
Soybean			26.00	21.00			Linear	Fletcher *et al.* (2007)
Soybean	2.13	—	30.80	—	−5.60	—	Segmented	
Soybean			26.11	13.62			Linear	Sadok and Sinclair (2009a)
Soybean	2.19	1.10	57.64	18.87	13.86	4.73	Segmented	
Soybean			29.73	20.17			Linear	Sadok and Sinclair (2009b)
Soybean	2.17	1.94	31.31	22.92	9.51	2.70	Segmented	
Wheat			40.90	34.40			Linear	Schoppach and Sadok (2012)
Wheat	3.89	2.40	60.80	39.30	31.20	3.10	Segmented	
Wheat	2.35	1.86	83.80	32.60	23.30	−3.30	Segmented	Schoppach *et al.* (2017)
Wheat			52.30	14.20			Linear	Tamang *et al.* (2019)
Wheat	2.80	1.90	83.80	21.90	24.40	−12.40	Segmented	
Wheat			61.72	30.52			Linear	Tamang *et al.* (2022)
Wheat	2.75	2.11	76.52	36.71	36.24	−18.96	Segmented	

The response is characterized by the coefficients of the (segmented) linear regression, i.e. the slope of the first line segment or the slope of the linear response (slope 1), the slope of the second line segment (slope 2), and the VPD where the two line segments intersect (VPD_BP_).

The transpiration rate response to decreasing soil moisture has also been investigated substantially (Table 2). Soil moisture can be expressed as water content (θ, vol. %; J. Zhang *et al*., 2021) or soil matric potential (ψ_soil_ or *h*_soil_, hPa or cm). Alternatively, the soil moisture level is expressed as the fraction of transpirable soil water (FTSW), defined as the percentage of the range of max. and min. soil/pot water holding capacity (Ritchie, 1973):

**Table 2.** Examples of studies reporting crop inter- and intra-specific variation for the transpiration rate response to decreasing soil moisture (SM)

Species	SM_BP_ min.	SM_BP_ max.	SM indicated as	Unit	Reference
Chickpea	0.46	0.41	FTSW	—	Anbazhagan *et al.* (2015)
Chickpea	0.58	0.57	FTSW	—	Pang *et al.* (2017)
Chickpea	0.86	0.44	FTSW	—	Pushpavalli *et al.* (2015)
Chickpea	0.63	0.30	FTSW	—	Zaman-Allah *et al.* (2011)
Maize	0.59	0.34	FTSW	—	Choudhary *et al.* (2020)
Maize	0.60	0.37	FTSW	—	Gholipoor *et al.* (2013b)
Maize	0.38	0.31	FTSW	—	Ray *et al.* (2002)
Maize	−180.00	−6.00	ψ_soil_	kPa	Cai *et al.* (2021)
Maize	−124.00	−16.00	ψ_soil_	kPa	Koehler *et al.* (2022)
Maize, soybean	0.37	0.27	FTSW	—	Ray and Sinclair (1998)
Peanut	0.67	0.30	FTSW	—	Bhatnagar-Mathur *et al.* (2007)
Peanut	0.47	0.40	FTSW	—	Devi and Sinclair (2011)
Peanut	0.71	0.22	FTSW	—	Devi *et al.* (2009)
Peanut	0.44	0.36	FTSW	—	Shekoofa *et al.* (2013)
Pearl millet	0.57	0.29	FTSW	—	Choudhary *et al.* (2020)
Pearl millet	0.49	0.30	FTSW	—	Kholová *et al.* (2010a)
Sorghum	0.48	0.38	FTSW	—	Choudhary *et al.* (2013a)
Sorghum	0.47	0.41	FTSW	—	Choudhary *et al.* (2013b)
Sorghum	0.53	0.32	FTSW	—	Choudhary *et al.* (2020)
Sorghum	0.48	0.32	FTSW	—	Gholipoor *et al.* (2012)
Soybean	0.55	0.47	FTSW	—	Devi *et al.* (2014)
Soybean	0.29	0.22	FTSW	—	Hufstetler *et al.* (2007)
Soybean	0.32	0.25	FTSW	—	Sadok *et al.* (2012)
Soybean	0.67	0.21	FTSW	—	Seversike *et al.* (2014)
Soybean	0.29	0.16	FTSW	—	Sinclair *et al.* (1998)
Soybean, cowpea, black gram, pigeon pea	0.30	0.20	FTSW	—	Sinclair and Ludlow (1986)
Wheat	0.52	0.38	FTSW	—	Itam *et al.* (2021)

The response is characterized by the soil moisture threshold (SM_BP_), upon which the transpiration rate decreases during soil drying.


$$\begin{array}{l} \text{FTS}{\text{W}}_{n}=\;\frac{\left(\text{P}{\text{W}}_{n\;}-\;\text{P}{\text{W}}_{\text{final}}\right)}{\left(\text{P}{\text{W}}_{\text{initial}}-\text{P}{\text{W}}_{\text{final}}\right)}, \end{array}$$ (4)

where FTSW_*n*_ is the fraction of transpirable soil water per day, PW_*n*_ (g) is the pot weight of each day over the experimental period, PW_final_ (g) is the pot weight by the end of the dry-down experiment, typically when the normalized transpiration ratio (NTR; see definition below) reaches 0.1, and PW_initial_ (g) is the pot weight at the beginning of the experiment when the soil is expected to be at maximum water holding capacity. The advantage of this expression is that it enables the comparison of plants grown in various soil textures with differing max. and min. soil moisture ranges.

Similarly, the transpiration rate is usually normalized to minimize the interference of day-to-day variations in environmental conditions (photosynthetically active radiation, vapor pressure deficit) and to be able to compare different-sized plants. This is done either by normalizing the transpiration rate by leaf area (e.g. Cai *et al.*, 2022b) or by a double normalization (normalized transpiration ratio [NTR]) of the daily transpiration rate value (*T*_*n*_, g) firstly, by dividing it by the daily average transpiration rate of the control pots per genotype in well-watered conditions (mean *T*_*n*,wet_, g):


$$\begin{array}{l} \text{T}{\text{R}}_{n}=\;\frac{T_{n}}{\text{mean }T_{n,\text{wet}}}\;, \end{array}$$ (5)

and secondly by dividing the resulting daily transpiration ratio (TR_*n*_) by the initial average transpiration ratio (mean TR_initial_) for each pot over the time when the respective pot was still in well-watered conditions (Devi *et al.*, 2010):


$$\begin{array}{l} \text{NT}{\text{R}}_{n}=\;\frac{\text{T}{\text{R}}_{n}}{\text{mean T}{\text{R}}_{\text{initial}}}\;. \end{array}$$ (6)

**Fig. 2.**
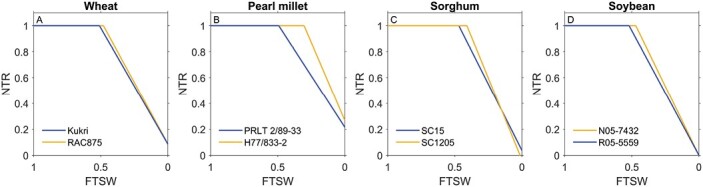
Characteristic response of normalized transpiration rate ratio (NTR) to decreasing fraction of transpirable soil water (FTSW) comparing a genotype that decreases transpiration rate at lower FTSW (i.e. in drier soil conditions, orange) versus a genotype that decreases transpiration rate at comparatively higher FTSW (i.e. in wetter soil conditions, blue). Redrawn for: wheat [Schoppach and Sadok, 2012, with permission from Elsevier, © Elsevier (2012), please note, the Open Access licence covering this article does not apply to this image]; pearl millet (Kholová *et al.*, 2010a); sorghum [Choudhary *et al.*, 2013b, with permission conveyed through Copyright Clearance Center, Inc., © CSIRO Publishing (2013), please note, the Open Access licence covering this article does not apply to this image]; and soybean [Devi *et al.*, 2014; © John Wiley and Sons (2104), please note, the Open Access licence covering this article does not apply to this image], with permission, using WebPlotDigitizer (Rohatgi, 2022).

As illustrated in Fig. 2, typical results report that the transpiration rate stays constant until a certain threshold soil moisture level (fraction of transpirable soil water breakpoint, FTSW_BP_), upon which the transpiration rate decreases almost linearly during further soil drying. Significant intra-specific variability exists in this value (Table 2). While some sensitive genotypes close stomata and reduce transpiration rate already in rather wet soil conditions (blue curves, Fig. 2), others might be able to still transpire maximally in comparatively dry soil conditions (yellow curves, Fig. 2).

It is worth mentioning that both types of experiments are typically conducted in pots in climate-chamber or greenhouse settings. Experiments on the field scale in a comparatively systematic manner have yet to be undertaken.

## Understanding the biophysical mechanisms underlying the transpiration rate response to increasing vapor pressure deficit and soil drying

### Transpiration rate response to increasing vapor pressure deficit

Sinclair *et al*. (2008b) and later Sadok and Sinclair (2010) proposed a link between plant hydraulic conductance and VPD_BP_. In soybean (*Glycine max* (L.) Merr), they found that a comparatively low plant hydraulic conductance was associated with a restricted transpiration rate. They proposed the limiting hydraulic conductance to be located between the xylem and the guard cells, specifically in the symplastic water pathway, potentially involving transmembrane water channels (i.e. aquaporin; Sadok and Sinclair, 2010; Schoppach and Sadok, 2012). In that case, a low plant hydraulic conductance would limit the water flux to the leaves, presumably resulting in a drop in leaf water potential triggering stomatal closure whenever water flow is insufficient to meet the transpiration rate under high-evaporative conditions (Bunce, 2006). It should be emphasized that while this explanation is sufficient to elucidate the extensively documented relationship between leaf water potential and stomatal conductance during atmospheric drying, stomatal physiological functioning is much more complex (e.g. considering guard cell hydromechanics and ABA dynamics). Nonetheless, also Choudhary *et al.* (2013a) and Jafarikouhini *et al.* (2020) found that a low leaf hydraulic conductance was associated with a limited transpiration rate under high VPD conditions in sorghum (*Sorghum bicolor* L.) and maize (*Zea mays* L. saccharata), respectively. Ocheltree *et al.* (2014) showed that a decreased stomatal conductance at increasing VPD was related to low plant hydraulic conductance in 19 grass species, more precisely to a low leaf hydraulic conductance. This is not surprising considering that leaves account for at least 30% of the hydraulic resistance within plants under well-watered conditions (Sack and Holbrook, 2006).

Choudhary *et al*. (2014) identified a low hydraulic conductivity in leaves *and* roots in maize (*Zea mays* L.). Similarly, Schoppach *et al.* (2014) found that a restricted transpiration rate at high VPD might be related to the hydraulic resistance in the roots of bread wheat plants (*Triticum aestivum* L.). They used different AQP inhibitors that were fed to de-rooted and intact plants. De-rooted plants did not show differences in VPD response or variations in transpiration sensitivity to AQP inhibitors between genotypes, contrary to intact plants. Moreover, genotypes differed in root hydraulic conductance when pressurizing the whole root system. Schoppach *et al.* (2014) concluded that the root system of VPD-responsive genotypes contributed to hydraulic limitation through a lack of AQP and smaller metaxylem vessels. The potential involvement of AQP in root hydraulic regulation and the connection to the transpiration rate response to increasing VPD was also reported by Kudoyarova *et al.* (2011) for durum wheat (*Triticum durum* Desf.), by Tharanya *et al.* (2018) for pearl millet (*Pennisetum glaucum* (L.) R.Br.), and by Sivasakthi *et al.* (2020) for chickpea (*Cicer arietinum* L.), for example. The suggested link between metaxylem vessel size and VPD responsiveness is in line with the results of Richards and Passioura (1989) for wheat. Note that the transpiration rate response to increasing VPD being either related to limitations in root hydraulic conductance or to restrictions in leaf hydraulic conductance does not necessarily posit a contradiction. A low root conductance will cause a more negative leaf water potential. In other words, a root hydraulic limitation will induce an earlier and more severe leaf hydraulic limitation in the water supply.

**Fig. 3.**
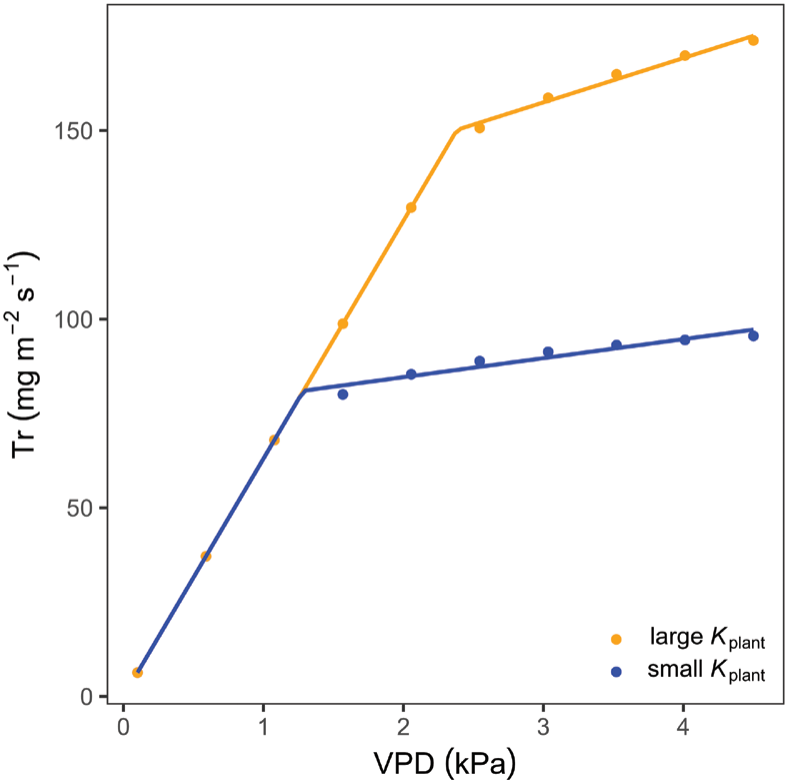
Theoretical relationship between transpiration rate (Tr) and vapor pressure deficit (VPD) in wet soil conditions (ψ_soil_=−100 hPa) comparing a genotype with a high plant hydraulic conductance (*K*_plant_, orange) and a genotype with a limited plant hydraulic conductance (blue). Based on modified parameterization of the model of Wankmüller and Carminati (2022). Tr becomes restricted at lower VPD when *K*_plant_ is small.

Hence, experimental evidence suggests that differences between genotypes in the transpiration rate response to increasing VPD are controlled by plant hydraulic conductance (Sinclair *et al.*, 2008b, 2017). We used the conceptual model of Wankmüller and Carminati (2022) to illustrate the response of Tr to increasing VPD for plants with contrasting plant hydraulic conductance (*K*_plant_, cm^3^ s^−1^ MPa^−1^). The model calculates the leaf water potentials based on transpiration rate, soil matric potential, and the hydraulic conductances of the components of the SPAC (a detailed description of the model can be found in Carminati and Javaux (2020) and Wankmüller and Carminati (2022)). The relationship between leaf water potential and gas exchange informs the stomatal regulation model, which is based on optimizing the carbon assimilation rate (*A*, μmol m^−2^ s^−1^) to leaf water potential ratio. The simulation predicts that plants with a lower *K*_plant_ are more sensitive to increasing VPD (blue curve, Fig. 3) in the typical setting of this experiment (i.e. in well-watered conditions), meaning that plants with a lower *K*_plant_ restrict Tr at lower VPD. In these conditions, the drop in water potential mainly occurs within the plant. A low plant (leave and/or root) hydraulic conductance causes a more negative leaf water potential to sustain a given transpiration rate and it triggers stomatal closure.

Taken together, growing experimental evidence concordantly indicates that plant traits and ecophysiological mechanisms that potentially modify the plant hydraulic conductance impact the transpiration rate response to increasing VPD. Such mechanisms include the abundance and activity of AQP in leaves (Kholová *et al.*, 2010b; Sadok and Sinclair, 2010; McAdam *et al.*, 2016) as well as in the stem (Shatil-Cohen *et al.*, 2011; Pantin *et al.*, 2013) and roots (Schoppach *et al.*, 2014; Tharanya *et al.*, 2018; Sivasakthi *et al.*, 2020; Sinclair and Jafarikouhini, 2022); leaf area expansion (timing, size, and exposure, e.g. Kholová *et al.*, 2010b; Zaman-Allah *et al.*, 2011; Sivasakthi *et al.*, 2017); xylem vessel size and abundance (Richards and Passioura, 1989; Comas *et al.*, 2013; Schoppach *et al.*, 2014); and stomatal properties (density and size, e.g. Xu and Zhou, 2008; Casson and Hetherington, 2010; Devi and Reddy, 2018). It is important to mention that besides traits impacting plant hydraulic conductance, the sensitivity to hormonal signals (e.g. ABA and IAA) plays a vital role in the behavior of transpiration rate under increasing transpiration demand (Kholová *et al.*, 2010b; McAdam *et al.*, 2015, 2016; Sussmilch and McAdam, 2017; Sadok and Schoppach, 2019). The interaction between hydraulic and non-hydraulic signals for water use regulation was recently discussed in greater detail in a review by Monnens and Sadok (2020).

### Transpiration rate response to soil drying

Compared with the transpiration rate response to increasing VPD, much less is known about the underlying mechanisms and plant hydraulic properties impacting the transpiration rate response to progressive soil drying. Evidence from Sinclair (2005) suggested that the transpiration rate response to soil drying is related to the loss in soil hydraulic conductivity with soil drying. This was supported by Sinclair *et al.* (2008a), who conducted a dry-down experiment with soybean in combination with applying hydrostatic pressure in the soil (in pressure pots) that is required to maintain leaf xylem water potential at zero with decreasing FTSW. Within this experimental set-up, they found that a small, relatively constant hydrostatic pressure had to be applied to the soil to maintain leaf xylem water potential at zero until a critical FTSW, which was followed by an increasing pressure gradient with decreasing FTSW and decreasing transpiration rate despite pressurization. The authors concluded that this behavior is mechanically linked to the gradients in hydrostatic pressure (or more generally, in matric potential) in the soil under drying conditions.

To understand the transpiration rate response to soil drying, relating transpiration rate to soil matric potential is as important as relating transpiration rate to volumes of water because volumetric water contents do not evenly translate into water accessibility to plants (Schweiger *et al.*, 2022, Preprint). While the concept of FTSW successfully unifies plant responses across soil textures, analysing transpiration rate as a function of soil matric potential provides insights into the factors and mechanisms driving soil water limitation for varying soils. Pressure differences in soil matric potential drive water flow and quantify the force a plant would have to apply to extract a unit of water from the soil. The matric potential accounts for capillary and adsorptive forces (Tuller and Or, 2001). For coarse-textured soils, it is equivalent to the hydrostatic pressure. In contrast, in fine-textured soils, adsorptive forces become relevant, and it is more comprehensive to express this component of the soil water potential as matric potential. Therefore, soil matric potential was proposed to link the soil’s water status with the plant’s water status (de Swaef *et al*., 2022). The relationship between transpiration rate and soil matric potential is not unique (Cai *et al.*, 2021; Koehler *et al.*, 2022) and differs between soil textures.

Building on Sperry and Love (2015) and Sperry *et al.* (2016), the soil–plant hydraulic framework of Carminati and Javaux (2020) argues that the development of matric potential gradients around the roots (and therefore the drop in soil hydraulic conductivity) is the primary constraint on transpiration rate under soil drying. During soil drying, soil hydraulic conductivity drops by several orders of magnitude. The drop in conductance is associated with an enhanced water depletion near the roots due to the radial nature of water flow into the roots, which causes a high water flux in close proximity to the roots (Gardner, 1965). Hence, under soil drying, plants experience an extremely fast matric potential loss when the soil becomes hydraulically limiting. It was proposed that stomata close when the matric potential around the roots drops more rapidly than the increase in transpiration rate in the context of soil drying (Carminati and Javaux, 2020), which is consistent with the analysis of Sinclair *et al.* (2008a). Melo *et al.* (2023) used a similar approach to estimate plant available water for varying soil textures. Recent studies have confirmed that the loss in belowground hydraulic conductivity (soil, roots, and/or soil–root interface) represents the primary driver of stomatal closure and gas exchange in drying soils (Rodriguez-Dominguez and Brodribb, 2020; Abdalla *et al.*, 2021, 2022).

**Fig. 4.**
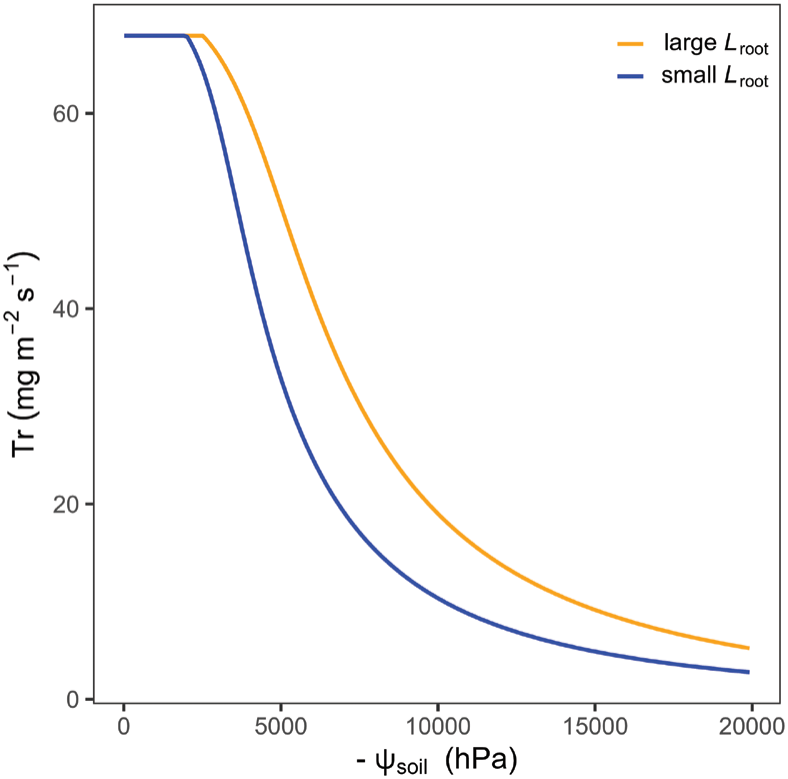
Theoretical relationship between transpiration rate (Tr) and soil matric potential (ψ_soil_) in low VPD conditions (VPD=1 kPa) comparing a plant with an extensive root system (*L*_root_, orange) and a plant with a small root system (blue). Based on modified parameterization of the model of Wankmüller and Carminati (2022). Tr drops at less negative ψ_soil_ (i.e. in wetter soil) when *L*_root_ is small.

To illustrate the importance of root and soil hydraulic properties for the transpiration rate response to soil drying, we used the model of Wankmüller and Carminati (2022) to simulate the response of Tr to decreasing soil moisture (here expressed as soil matric potential, ψ_soil_, hPa) for two plants exhibiting differential active root length (*L*_root_, cm). The rationale is that the size of the root system actively taking up water is a critical determinant for the water fluxes and soil matric potential gradients at the soil–root interface during soil drying because a bigger active root length means that the transpiration rate-induced fluxes are distributed over a larger surface, which results in lower fluxes (cm s^−1^) in the soil. A more extensive root system would attenuate soil matric potential gradients at the soil–root interface and thereby slow down the water flux in the soil (Faiz and Weatherley, 1982). Therefore, plants with a larger root system are expected to maintain transpiration rate at comparatively lower soil matric potentials (Abdalla *et al.*, 2022). Note that we refer to the active root length here rather than to the total root length since it was shown that not all roots are equally active in water uptake (Ahmed *et al.*, 2016, 2018). Indeed, the simulation outcome suggests that a plant with a smaller root system decreases transpiration in wetter soil conditions (i.e. less negative ψ_soil_, blue curve, Fig. 4), in consequence of the large drop in soil hydraulic conductivity to sustain the transpiration demand.

**Fig. 5.**
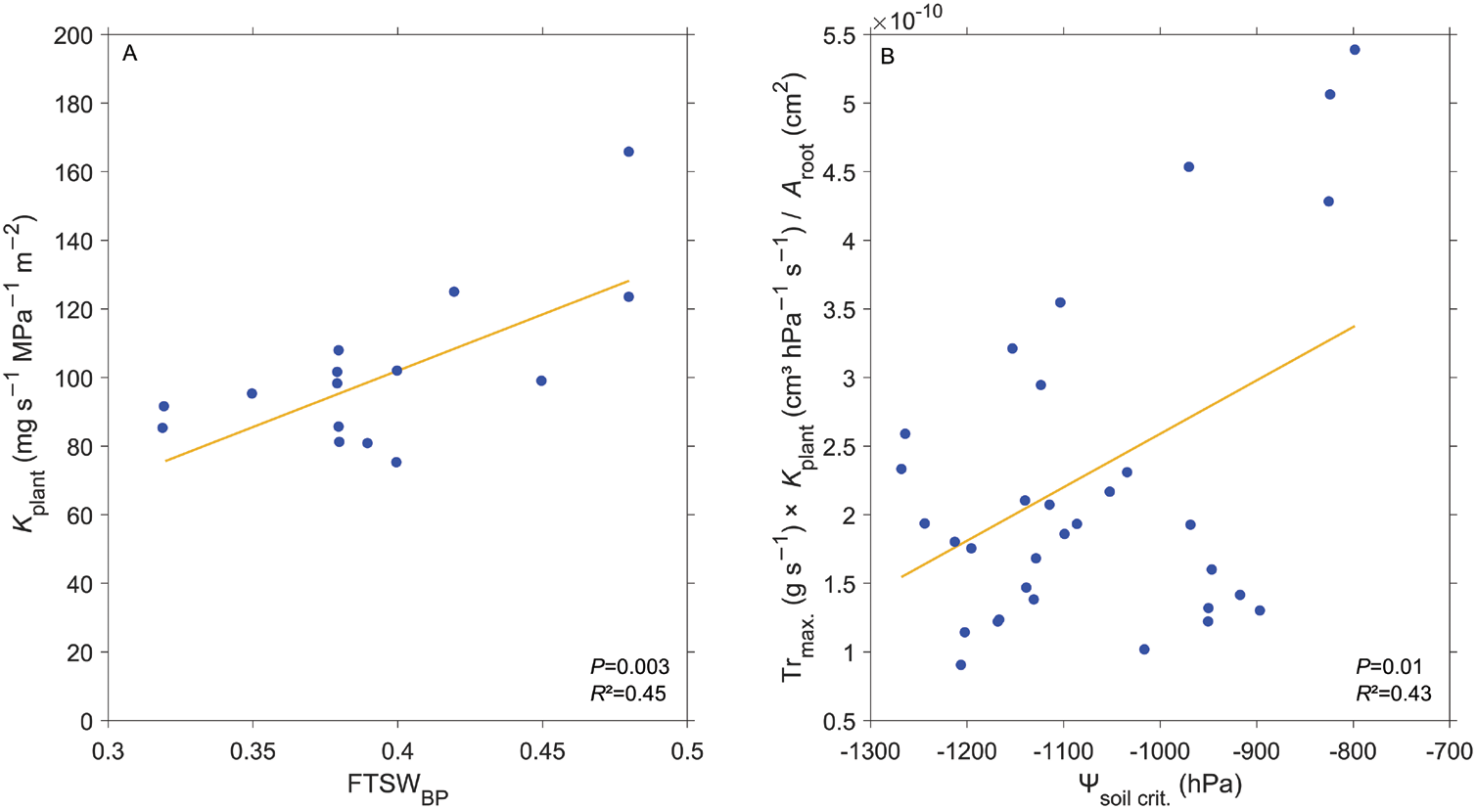
Relationship between plant traits that are expected to impact the development of matric potential gradients around the roots during soil drying and the critical soil moisture level upon which plants decrease transpiration rate in drying soil. (A) The plant hydraulic conductance (*K*_plant_) and the fraction of transpirable soil water (FTSW) breakpoint (FTSW_BP_). Redrawn using WebPlotDigitizer (Rohatgi, 2022) from Choudhary and Sinclair (2014), with permission conveyed through Copyright Clearance Center, Inc., © CSIRO Publishing (2014), please note, the Open Access licence covering this article does not apply to this image. (B) A combination of variables that impact the development of water potential gradients around the roots during soil drying: maximum transpiration rate (Tr_max_, i.e. plant water demand), plant hydraulic conductance (*K*_plant_), and the root surface area that actively takes up water (*A*_root_, i.e. plant water supply); and the critical soil matric potential (ψ_soil,crit_). Redrawn using WebPlotDigitizer (Rohatgi, 2022) from Koehler *et al.* (2023), by permission of Oxford University Press, © Oxford University Press (2023), please note, the Open Access licence covering this article does not apply to this image. In both studies, a higher plant hydraulic conductance was associated with an earlier (i.e. in relatively wet soil conditions) decrease in transpiration rate.

While it is accepted that the transpiration rate response to soil drying is linked to a decrease in soil (or generally belowground) hydraulic conductivity, how plant hydraulic properties impact this response still needs to be resolved. Currently, two contradictory concepts have been identified. The first concept posits that genotypes decreasing transpiration rate in comparatively wetter soil conditions are characterized by a low plant/root hydraulic conductance (Belko *et al.*, 2012; Gholipoor *et al.*, 2012, 2013b; Shekoofa *et al.*, 2013). The low plant conductance causes the plant to reach critical leaf water potentials at less negative soil matric potentials (i.e. in wetter soils). In this case, the ‘water-saving’ behavior during atmospheric drying (i.e. decreasing Tr at lower VPD daily) induced by a low plant hydraulic conductance is extended to the case of soil drying on a seasonal basis. The alternative concept points to the opposite: genotypes decreasing transpiration rate in comparatively wetter soil conditions are characterized by a higher plant/root hydraulic conductance. Experimental evidence supports this counterintuitive idea: Choudhary and Sinclair (2014) found that sorghum (*Sorghum bicolor* L.) genotypes that decreased transpiration rate at higher FTSW (i.e. less negative ψ_soil_) are expressing a comparatively higher plant conductance (Fig. 5A). This was also shown by Cai *et al.* (2022a) for several plant species (namely: wheat, barley, maize, and tomato) and Koehler *et al.* (2023) for maize (Fig. 5B). Note that in Fig. 5B, Koehler *et al.* (2023) included the maximum transpiration (as a measure of plant water demand) divided by root surface area (as a measure of plant water supply), besides the plant hydraulic conductance, as a factor determining the onset of soil hydraulic limitations. The reasoning behind the counterintuitive relation between plant hydraulic conductance and ψ_soil_ is explained in the following. Considering the SPAC as a system of hydraulic resistances in series, meaning that the overall resistance is equal to the sum of the single resistances of the compartments of the SPAC, the plant hydraulic resistance (i.e. the inverse of the hydraulic conductance) will determine how susceptible a plant is to a change in total conductance. For plants with low hydraulic resistance (i.e. a high hydraulic conductance), the soil hydraulic resistance will become a limiting factor for the overall resistance sooner in such a system in series, and plants will sense it earlier. Plants with a high conductance are therefore expected to be more sensitive in their transpiration rate response to an increase in matric potential gradients around roots in drying soils. Note that this concept implies that plants, especially stomata, respond to a change in soil–plant hydraulic conductance rather than to an absolute leaf water potential.

Despite the contradictions regarding the role of the plant hydraulic conductance in the transpiration rate response to soil drying, traits and processes that are suggested to impact this response generally relate to properties that influence the development of matric potential gradients around the roots (i.e. belowground hydraulic conductance) during soil drying. These include processes that modify the root surface area that actively takes up water, which depends on root architectural traits (Doussan *et al.*, 2006; Lynch, 2013), mechanisms of hydraulic regulation in interaction with hormonal signaling (e.g. root AQP activity/turnover (Knipfer *et al.*, 2011; Chaumont and Tyerman, 2014; Tardieu *et al.*, 2017; Reddy *et al.*, 2022) as impacted by, for example, ABA, as discussed in the previous sections), and anatomical traits, e.g. root xylem vessel size and abundance (Frensch and Steudle, 1989; Richards and Passioura, 1989; Strock *et al.*, 2021), root cortical cell size (Chimungu *et al*., 2014a, b), and root cortical aerenchyma (Zhu *et al.*, 2010; Chimungu *et al.*, 2015). Finally, traits and processes that shape the rhizosphere hydraulic conductance during soil drying, e.g. root hair formation (Carminati *et al.*, 2017; Marin *et al.*, 2021; Duddek *et al.*, 2022), mycorrhizal fungal association (Augé, 2001; Vidal *et al.*, 2018; Abdalla and Ahmed, 2021), and mucilage exudation (Carminati *et al.*, 2010; Ahmed *et al.*, 2014), are expected to impact the gradients in matric potential around the roots and thus the onset of hydraulic limitations.

## Synthesis: combined response to high vapor pressure deficit and low soil moisture

**Fig. 6.**
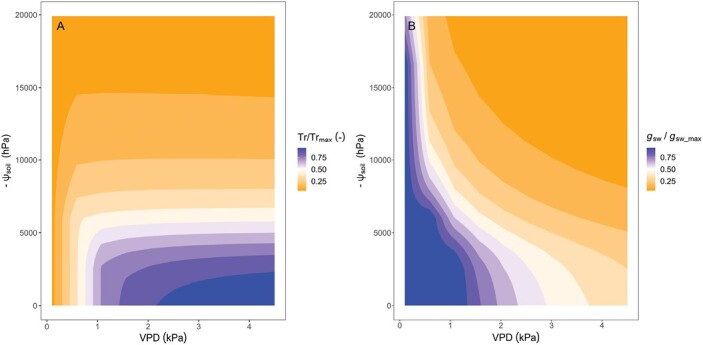
Heat map of the theoretical response of transpiration rate (Tr, A) and stomatal conductance (*g*_sw_, B) to decreasing soil matric potential (ψ_soil_) combined with increasing vapor pressure deficit (VPD) as the two major environmental constraints on leaf-level gas exchange. Based on modified parameterization of the model of Wankmüller and Carminati (2022). High values (blue hues) indicate no or slight hydraulic limitation on Tr and *g*_sw_. Low values (orange hues) indicate a strong hydraulic limitation on Tr and *g*_sw_. At less negative ψ_soil_, the transpiration rate can be sustained until comparatively high VPD (A), while at more negative ψ_soil_ stomata close at relatively low VPD (B). At low VPD, transpiration rate can be sustained even at more negative ψ_soil_ (A), while at high VPD, stomata partially close even at less negative ψ_soil_ (B).

The transpiration rate response to increasing VPD and to decreasing soil moisture is often experimentally investigated separately, but at least under certain water availability regimes, they interact. The transpiration rate response to VPD is exacerbated by soil drying (meaning that plants would restrict transpiration rate at comparatively lower VPD in drier soil; e.g. Cai *et al.*, 2022b). Similarly, the transpiration rate response to soil drying is exacerbated by increasing VPD (meaning that plants would decrease the transpiration rate at relatively higher soil moisture levels if the VPD is high; e.g. Devi and Reddy, 2020; P. Zhang *et al.*, 2021, 2022). These results are easily predicted using the water demand–supply framework, as that implemented in Wankmüller and Carminati (2022), and illustrated in Fig. 6: (i) at high soil moisture levels (less negative ψ_soil_), the transpiration rate can be sustained until comparatively high VPD (Fig. 6A), while at low soil moisture (more negative ψ_soil_) stomata close at relatively low VPD (Fig. 6B); and (ii) at low VPD, transpiration rate can be sustained even in relatively dry soils (more negative ψ_soil_, Fig. 6A), while at high VPD, stomata partially close even when the soil is still rather wet (at less negative ψ_soil_, Fig. 6B).

**Fig. 7.**
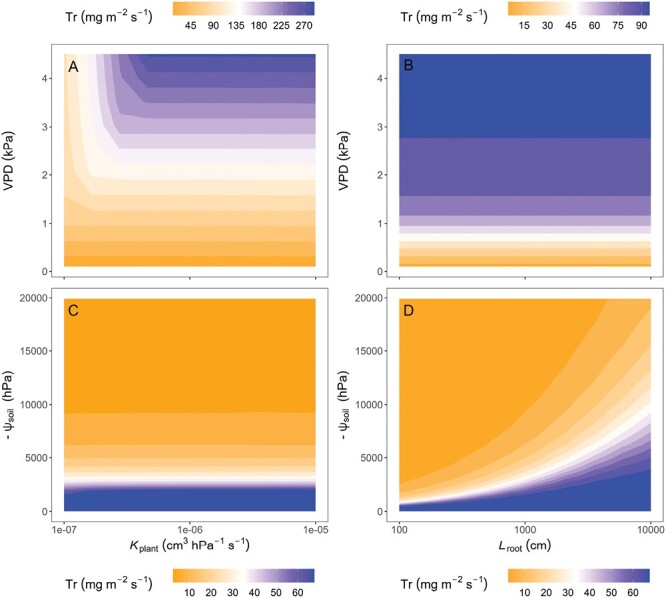
Heat map of the theoretical response of transpiration rate (Tr) to (A) vapor pressure deficit (VPD) as dependent on the expression of the plant hydraulic conductance (*K*_plant_), (B) VPD as dependent on root length (*L*_root_), (C) soil matric potential (ψ_soil_) as dependent on the expression of the plant hydraulic conductance (*K*_plant_), and (D) ψ_soil_ as dependent on root length (*L*_root_). Based on modified parameterization of the model of Wankmüller and Carminati (2022). The sensitivity of the transpiration rate to increasing VPD is high for traits that impact the hydraulic conductance of the plant (e.g. *K*_plant_, A) rather than for traits that affect the water flow from the soil to the roots (e.g. *L*_root_, B). During soil drying, plants are susceptible to traits that modify the water flow in the soil (e.g. *L*_root_, D) rather than to traits that affect the plant internal water flow (e.g. *K*_plant_, C).

Atmospheric and soil drying are interlinked by their impact on the hydraulic conductance of the SPAC. Hence both examined drivers of plant water deficit have similar effects on decreasing the transpiration rate. However, hydraulic constraints arise in different parts of the SPAC during atmospheric drying versus soil drying. Therefore, plant hydraulic traits that control plant water use regulation may vary with the two environmental drivers. We used the conceptual framework of Wankmüller and Carminati (2022) to simulate the transpiration rate response to atmospheric and soil drying in dependence on traits that will either predominantly impact water flow through the plant (e.g. *K*_plant_) or the soil (e.g. *L*_root_) to identify traits relevant in different environments. During atmospheric drying, the transpiration rate response to increasing VPD is associated with hydraulic limitations within the plant tissues. Therefore, the sensitivity of the transpiration rate to increasing VPD is high for traits that impact the hydraulic conductance of the plant (e.g. *K*_plant_, Fig. 7A) rather than for traits that affect the water flow from the soil to the roots (e.g. *L*_root_, Fig. 7B). During soil drying, hydraulic limitations predominantly take place in the soil. Hence, plants are susceptible to traits that modify the water flow in the soil (e.g. active root length in relation to transpiring fraction of leaf area (Cai *et al.*, 2023), *L*_root_, Fig. 7D) rather than to traits that affect the plant internal water flow (e.g. *K*_plant_, Fig. 7C). Note that these considerations are based on pot experiments, where roots are uniformly distributed. Under field conditions, the root distribution over depth, and precisely the decline in root length density with increasing depth, is an additional determinant of the decline of transpiration during soil drying. Transpiration response to atmospheric drying and to soil drying has yet to be systematically investigated at the field scale and the gradients in root and soil water distribution over depth are crucial factors to be considered. To summarize, depending on the environmental scenario, plant properties predominantly affecting the transpiration rate response to increasing VPD and soil drying might differ. This awareness is essential to target environment-specific breeding for crop drought adaptation.

**Fig. 8.**
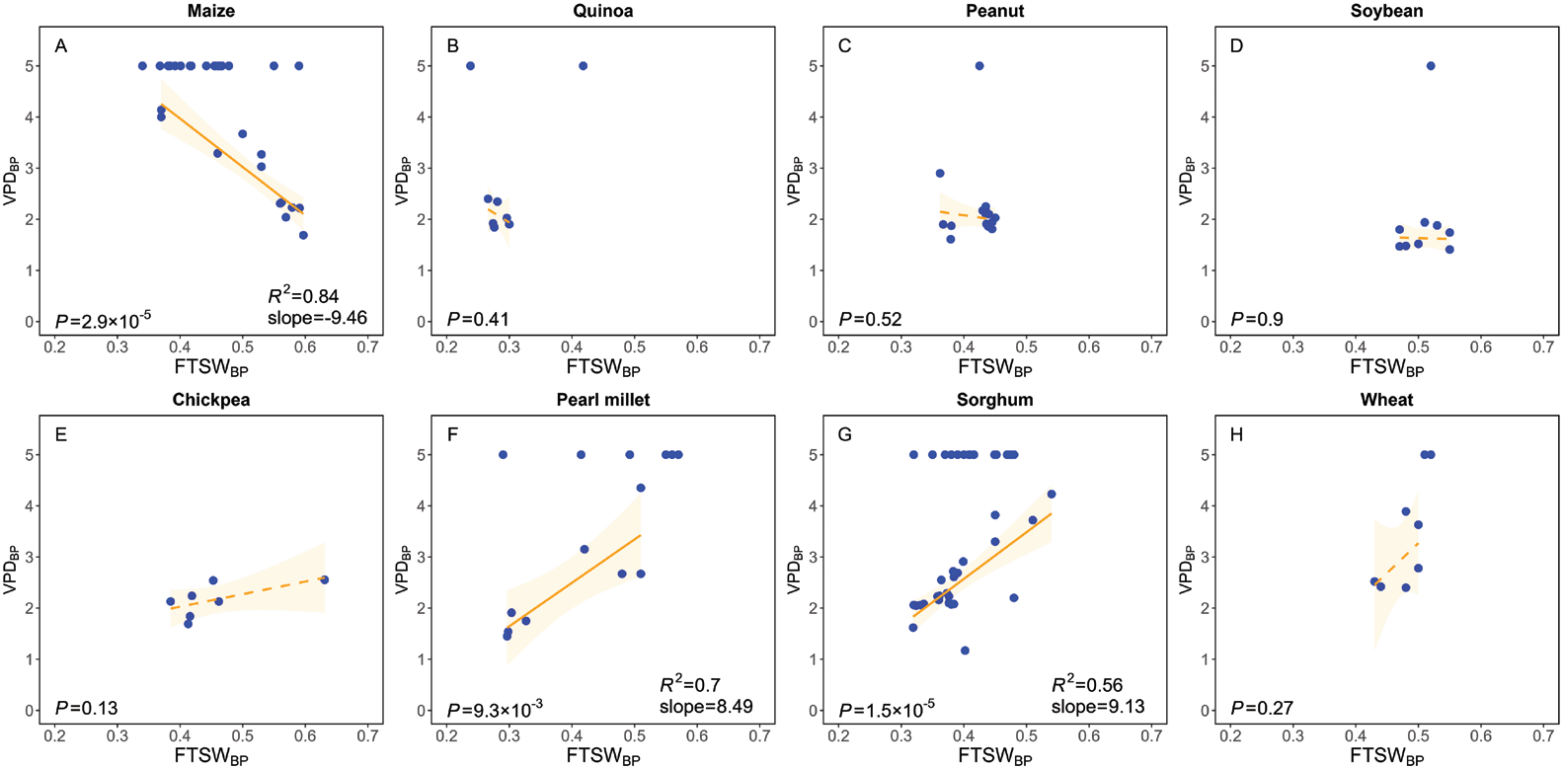
Relation between the fraction of transpirable soil water (FTSW) breakpoint (FTSW_BP_) upon which plant transpiration rate decreases in response to soil drying, and the vapor pressure deficit (VPD) breakpoint (VPD_BP_) upon which the increase in transpiration rate with increasing VPD is restricted for. (A) Maize (data from Gholipoor *et al.*, 2013a, b; Choudhary *et al.*, 2020). (B) Quinoa (data from Sanchez *et al.*, 2021). (C) Peanut (data from Devi and Sinclair, 2011; Shekoofa *et al.*, 2013). (D) Soybean (data from Devi *et al.*, 2014). (E) Chickpea (data from Zaman-Allah *et al.*, 2011; Anbazhagan *et al.*, 2015). (F) Pearl millet (data from Kholová *et al.*, 2010a, b; Choudhary *et al.*, 2020). (G) Sorghum (data from Gholipoor *et al.*, 2010, 2012; Choudhary *et al.*, 2013a, 2020). (H) Wheat (data from Schoppach and Sadok, 2012). Note that solid and dashed regression lines indicate significant and non-significant relations, respectively. The shaded area represents the 95% confidence interval.

Above, we analysed the transpiration rate sensitivity to two emerging plant properties (*K*_plant_ and *L*_root_) independently. However, to some extent, they might be positively correlated (Deguchi *et al.*, 2015; Meunier *et al.*, 2017; Cai *et al.*, 2022a). This implies that plant water use strategies (i.e. conservative versus consumptive) in response to increasing VPD and soil drying might be similar. Therefore, we compiled the results of studies that investigated the transpiration responsiveness to atmospheric drying (indicated by VPD_BP_) and the transpiration responsiveness to soil drying (indicated by FTSW_BP_) for crops. There seem to be three patterns. Firstly, within certain species, some genotypes appear to be either overall conservative (low VPD_BP_ and high FTSW_BP_) or consumptive (high VPD_BP_ and low FTSW_BP_) in their water use (e.g. maize, quinoa, peanut, and soybean, Fig. 8A–D). Secondly, there seem to be genotypes of species that are either conservative in their daily response to increasing VPD (low VPD_BP_ and low FTSW_BP_) *or* in their seasonal response to decreasing soil moisture, and vice versa (high FTSW_BP_ and high VPD_BP_, e.g. chickpea, pearl millet, sorghum, and wheat, Fig. 8E–H). Thirdly, there are genotypes within each species that show no VPD_BP_ but cover the full range of FTSW_BP_. The correlation is statistically significant for pearl millet, sorghum, and maize and hence considered to be indicative of trends worth discussing. We suggest those trends to be a function of plant water demand and supply in dependency on hydraulic traits. In the following, we attempt to explain the observed patterns.

**Fig. 9.**
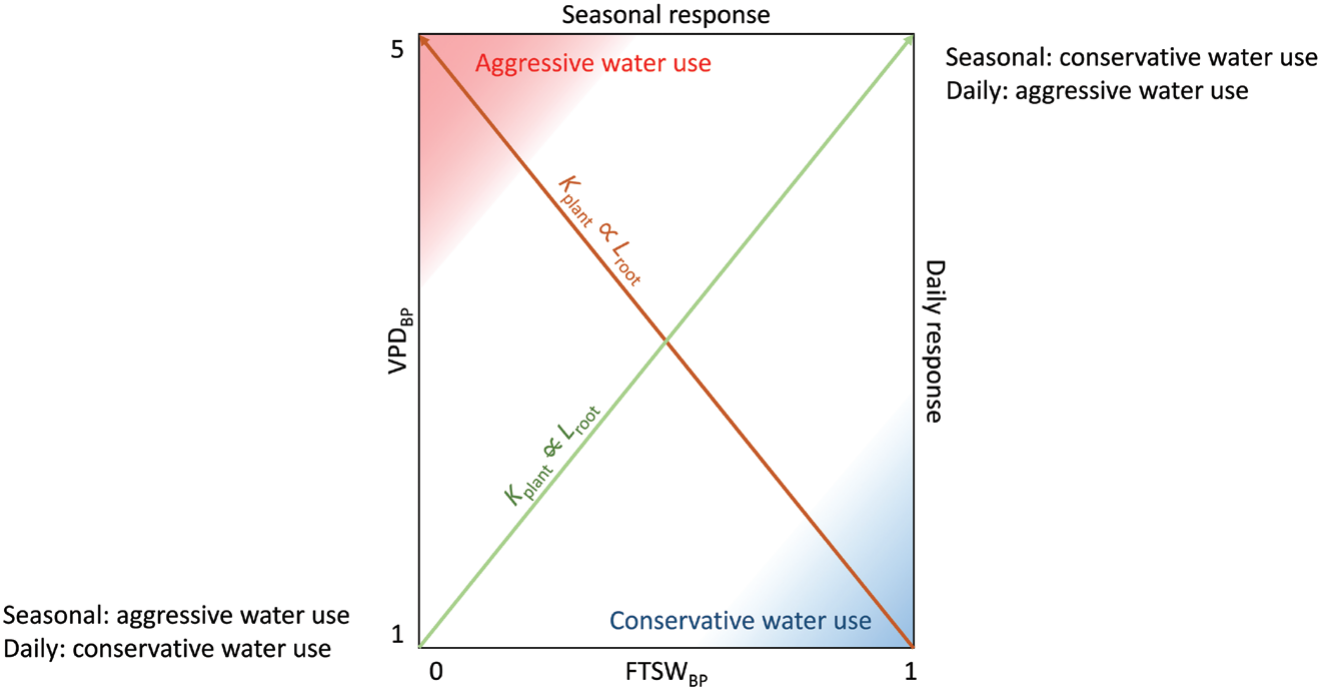
Theoretical range of relations between the fraction of transpirable soil water (FTSW) breakpoint (FTSW_BP_) upon which plant transpiration rate decreases in response to soil drying, and the vapor pressure deficit (VPD) breakpoint (VPD_BP_) upon which the increase in transpiration rate with increasing VPD is restricted.

The first pattern, where genotypes within certain species exhibit a range of water usage from conservative to consumptive on both daily *and* seasonal scales (moving on the brown line in Fig. 9), may not be intuitive. When the daily average transpiration rate is comparatively low due to a restricted transpiration rate at high VPD (e.g. at noon), plants would appear to sustain the reduced fluxes during soil drying more easily and longer. Hence, from a simple hydraulic perspective, a strong daily transpiration response to increasing VPD should lead to a conservative seasonal transpiration response to soil drying (moving along the green line in Fig. 9). However, plants with a high hydraulic conductance and extensive root systems may be able to maintain transpiration at increasing VPD and decreasing soil moisture (Fig. 9, upper left corner). On the other hand, plants with low internal conductance and limited root system extension will restrict transpiration at low VPD and experience critical gradients in soil matric potential in relatively wet soil conditions (Fig. 9, lower right corner). The aggressive/consumptive water use strategy, where transpiration is restricted only at high VPD and in dry soil conditions (Fig. 9, upper left corner), may be beneficial in environments with high evaporation rates to enable a rapid and efficient water use of soil moisture before it is ‘lost’ to evaporation (Schoppach and Sadok, 2012; Sadok *et al.*, 2019). However, this approach may be hazardous in environments with a high likelihood of prolonged drought or for plant varieties that take longer to mature, as it increases the risk of survival of the plant until it reaches a point where it can be used for agriculture. The conservative water use strategy, where transpiration is restricted at low VPD and high soil moisture levels (Fig. 9, lower right corner), may be beneficial in environments terminally exposed to water limitations as it enables a plant to save water needed for the grain filling period during less critical physiological times for agronomic performance (Sinclair and Muchow, 2001; Sinclair *et al.*, 2005, 2010; Messina *et al.*, 2015). However, such conservative genotypes might suffer from disadvantageous limitations on carbon assimilation when enough moisture is available to trade water loss from transpiration for CO_2_ uptake in moister environments. Genotypes of maize seem to significantly follow this pattern of being either wholly conservative or aggressively consumptive. Their water use is expected to be extremely sensitive to variations in hydraulic traits (e.g. *K*_plant_∝*L*_root_).

The second pattern, where genotypes within certain species exhibit a conservative or consumptive water use pattern either daily *or* seasonally (moving on the green line, Fig. 9), is more in line with what would be expected, as discussed above. Additionally, it may not be reasonable to assume that plant (root) hydraulic conductance is always proportional to root length. Vadez (2014), for example, suggested that plant (root) hydraulics rather than root length have a more significant impact on crop water use. In a plant with a low hydraulic conductance, independent of root length (*K*_plant_∝*L*_root_), stomata may close at relatively low VPD during atmospheric drying (e.g. Sinclair *et al.*, 2008b; Sadok and Sinclair, 2010; Choudhary *et al.*, 2014), leading to conservative water use on a daily scale (Fig. 9, lower left corner). In the case of decreasing soil water availability, the low plant hydraulic conductance would cause the plant to be less sensitive to a decline in soil hydraulic conductivity (Fig. 5, e.g. Choudhary and Sinclair, 2014; Koehler *et al.*, 2023), allowing for sustained transpiration rates at relatively lower soil moisture levels during soil drying (i.e. being consumptive on the seasonal scale; Fig. 9, lower left corner). On the other hand, a highly conductive plant would be less susceptible to daily water stress but more sensitive to seasonal water stress (i.e. during soil drying; Fig. 9, upper right corner). Being conservative in water use on a daily scale and consumptive on a seasonal scale may be beneficial in environments that frequently experience cyclic drought, as it allows for water conservation in the soil until the next precipitation event (Vadez *et al.*, 2014). Being consumptive or aggressive in water use on a daily scale and conservative on a seasonal scale might be beneficial for fast-maturing plants in environments commonly exposed to late-season drought, as it allows for continued carbon uptake. Crops such as pearl millet and sorghum follow this pattern significantly.

Lastly, some plants (across species and genotypes) do not show any restriction in transpiration rate when VPD increases (in Fig. 8 indicated by VPD_BP_ of 5 kPa). An increasing number of studies show that the consistent identification of VPD responsiveness is difficult as it interacts with the environmental conditions during the growth period. The exposure to a comparatively high average VPD over the growth period was shown to lead to a shift in the transpiration rate restriction to occur at higher VPD or even to the loss of the transpiration rate sensitivity to VPD (Yang *et al.*, 2012; Seversike *et al.*, 2013; Riar *et al.*, 2015; Choudhary *et al.*, 2020). The transpiration rate may not show restrictions when VPD increases due to a relatively high plant hydraulic conductance that can match the transpiration water flow (Tr) Thus, it never becomes a limiting factor, for instance when (Tr_max_/*K*_plant_)≥0.5 MPa, where Tr_max_ is the transpiration rate at the highest VPD. It was hypothesized that this transpiration rate behavior is especially beneficial in well-watered environments as it enables a plant to exploit the full carbon assimilation potential when there is no apparent risk of water deficit (Sadok *et al.*, 2019; Tamang *et al.*, 2019).

In summary, how plants respond to drought (by being conservative or consumptive in water use) is determined by the dynamics of the interaction between water demand (VPD) and supply (soil moisture availability) during the crop cycle, which determine the transpiration sensitivity to key hydraulic traits. Our species comparison suggests that species have different water use strategies and traits controlling their water use when exposed to atmospheric and soil drying, indicating different sources of hydraulic limitations and varying sensitivities to adjustments of traits.

## Conclusion

Experimental investigations of the transpiration rate response to increasing VPD and soil drying are often carried out independently to identify the hydraulic and physiological bottlenecks. When the atmosphere dries, water potential gradients within the plant are expected to limit water flow. Under soil drying, the development of water potential gradients in the soil and at the soil–root interface will trigger water flow limitations. Therefore, traits and physiological mechanisms involved in the transpiration rate response to atmospheric drying differ from the ones impacting soil drying. Hence, although the underlying mechanisms of transpiration regulation are similar (a drop in hydraulic conductance triggers stomatal closure), plant transpiration sensitivity to hydraulic traits can differ, resulting in different water use strategies daily and on seasonal time scales. This variability in plant water use strategies and their effects on crop productivity in water-limited regions have not yet been fully explored. That would require experiments exploring the interaction between rising VPD and soil drying. Moreover, investigating the effect of atmospheric drying and soil drying on plant transpiration at the field scale is still pending.

## Data Availability

The numerical code to generate Figs 3, 4, 6, and 7 is openly available in figshare at https://doi.org/10.6084/m9.figshare.23560659.v1.
